# Integrating sepsis management recommendations into clinical care guidelines for district hospitals in resource-limited settings: the necessity to augment new guidelines with future research

**DOI:** 10.1186/1741-7015-11-107

**Published:** 2013-04-18

**Authors:** Shevin T Jacob, Matthew Lim, Patrick Banura, Satish Bhagwanjee, Julian Bion, Allen C Cheng, Hillary Cohen, Jeremy Farrar, Sandy Gove, Philip Hopewell, Christopher C Moore, Cathy Roth, T Eoin West

**Affiliations:** 1 International Respiratory and Severe Illness Center (INTERSECT), Department of Medicine, University of Washington, 325 9th Ave, Box 359927, Seattle, WA, 98104, USA; 2 Department of Health and Human Services, Office of Global Affairs, 200 Independence Ave SW, Washington, DC, 20201, USA; 3 Community Health Department, Masaka Regional Referral Hospital, Masaka, Uganda; 4 Department of Anesthesiology and Pain Medicine, University of Washington, 1959 NE Pacific Street, BB-1469, Box 356540, Seattle, WA, 98195, USA; 5 University Department Anaesthesia & Intensive Care Medicine, N5, Queen Elizabeth Hospital (old site), Edgbaston, Birmingham, B15, 2TH, UK; 6 Department of Epidemiology and Preventive Medicine, Monash University, 99 Commercial Road, Melbourne, VIC, 3004, Australia; 7 Department of Emergency Medicine, Maimonides Medical Center, 4802 Tenth Avenue, Brooklyn, NY, 11219, USA; 8 The Hospital for Tropical Diseases, Oxford University Clinical Research Unit, 764 Vo Van Kiet Street, Ward 1, District 5, Ho Chi Minh City, Vietnam; 9 IMAI-IMCI Alliance, San Francisco, CA, USA; 10 Division of Pulmonary and Critical Care Medicine, Department of Medicine, San Francisco General Hospital, University of California, San Francisco, 1001 Potrero Avenue, San Francisco, CA, 94110, USA; 11 Division of Infectious Diseases and International Health, Department of Medicine, University of Virginia, PO Box 801340, Charlottesville, VA, 22908, USA; 12 Health Security and Environment Department, World Health Organization, 20 20 Avenue Appia, 1211, Geneva, 27, Switzerland

**Keywords:** Sepsis, Resource-limited setting, Severe illness, Guidelines

## Abstract

Several factors contribute to the high mortality attributed to severe infections in resource-limited settings. While improvements in survival and processes of care have been made in high-income settings among patients with severe conditions, such as sepsis, guidelines necessary for achieving these improvements may lack applicability or have not been tested in resource-limited settings. The World Health Organization’s recent publication of the Integrated Management of Adolescent and Adult Illness District Clinician Manual provides details on how to optimize management of severely ill, hospitalized patients in such settings, including specific guidance on the management of patients with septic shock and respiratory failure without shock. This manuscript provides the context, process and underpinnings of these sepsis guidelines. In light of the current deficits in care and the limitations associated with these guidelines, the authors propose implementing these standardized best practice guidelines while using them as a foundation for sepsis research undertaken in, and directly relevant to, resource-limited settings.

## Background

To provide guidance on a comprehensive approach to the management of children and adult patients with common diseases and conditions in resource-limited settings, the World Health Organization (WHO) has coordinated the development of a set of integrated management tools. These tools, comprised of guidelines and health worker training modules, include the Integrated Management of Childhood Illness, the Integrated Management of Pregnancy and Childbirth, and the Integrated Management of Adolescent and Adult Illness (IMAI). Many of these tools have been widely disseminated globally [1]. In July 2011, the WHO approved the publication of the most recent addition to the integrated management series, the IMAI District Clinician Manual (DCM). This document, just released on-line and in print, is a two-volume publication providing guidance on the care of hospitalized adult patients in district hospitals from resource-limited settings [2].

In resource-limited settings, management of severely ill patients is particularly challenging due to a difficult balance between a high caseload of patients and the correspondingly low supply of necessary medical equipment and human resources [3,4]. Public health crises such as the 2009 H1N1 influenza pandemic have reinforced concerns about the potential for a rapid overwhelming of the global health system’s capacity to manage patients with severe acute illness, particularly in settings with limited resources [5]. To address this challenge, the DCM emphasizes a practical and systematic approach to triage and management of all patients presenting to the hospital in resource-limited settings with an acute illness, including those who are critically ill.

Infectious diseases have a disproportionate impact on populations in low and middle income countries and cause correspondingly high mortality rates [6,7]. Regardless of the etiology, one of the most common pathways contributing to this mortality is the ‘sepsis syndrome’, defined by the presence of both infection and a systemic inflammatory response. Further along the severity spectrum of this syndrome, severe sepsis and septic shock can occur with increasing lethality resulting from progression to end-organ damage and refractory hypotension [8]. While it is poorly quantified, the global burden of sepsis is undoubtedly massive. Extrapolating from North American data, some 15 to 20 million cases of severe sepsis among adults occur worldwide each year, half of which may be fatal [9]. Available data on sepsis management of adults in resource-limited settings suggest that this high mortality is associated with ineffective management including delayed and improper empiric antimicrobial therapy as well as sub-optimal fluid resuscitation [10,11]. Thus, attention to reducing the mortality from sepsis by focusing on improved management in these settings is urgently needed.

Given the importance of this condition, in 2009 the WHO convened an international group of experts to review the current management of sepsis and to identify an evidence-based strategy and approach for sepsis management in resource-limited settings. These guidelines were subsequently included in the IMAI DCM as a framework for the management of severe acute illness in adults. The purpose of this article is to delineate the barriers to optimal sepsis management in resource-limited settings, describe the approach used to develop the IMAI DCM sepsis guidelines and their inherent limitations, identify the knowledge gaps and highlight key questions that must be answered to further inform and refine best practice for the management of sepsis in these settings.

### Current standard of care of sepsis in high-resource settings

Studies conducted in high-income countries have suggested a benefit from early sepsis identification coupled with targeted interventions in the management of patients with severe sepsis. These interventions include rapid administration of appropriate antimicrobial therapy, additional source control of infection as needed, and optimization of tissue perfusion using fluids, vasopressors and inotropes, and red blood cell transfusions. These initial actions are accompanied by support of failing organs and appropriate efforts to minimize complications. Protocol-driven strategies are the basis for the best practice guidelines for sepsis care promulgated by the International Surviving Sepsis Campaign [12]. Several independent, multi-site studies from middle and high income countries have demonstrated improved survival in parallel with improvements in compliance with aggregate best practice guidance (‘sepsis bundles’) [12-22]. Importantly, the results of studies re-evaluating the efficacy of certain sepsis bundle components (for example, activated protein C, tight glucose control and low-dose steroids) has led to revision, and sometimes removal, of these components in more recent iterations of the guidelines [23]. Thus, in an effort to independently evaluate goal-directed resuscitation in adults with septic shock, three parallel randomized-controlled multicenter trials in Australia, the USA and the UK are underway [24-26].

### Applicability of existing sepsis guidelines to resource-limited settings

While there is some evidence to support the feasibility of implementing modified sepsis management guidelines in resource-limited settings [27], substantial health system and resource challenges impede the translation of current best practice guidelines in these environments [28-31]. For example, variable access and long distances to hospitals contribute to severely ill patients presenting to the health system. In addition, triage capacity is often absent in district hospitals, leading to further delays in recognizing ill patients and initiating treatment. Once recognized, a lack of effective antimicrobials, oxygen and intravenous fluids may further impair provision of optimal sepsis care. Moreover, human resources are often scarce with respect to both quantity and expertise, thus precluding the labor-intensive iterative approach that characterizes sepsis management in high resource settings.

Identifying which should be the priority interventions for sepsis management in a resource-limited setting is a further challenge. While evidence exists for the benefit of prompt and appropriate antimicrobial therapy, answering questions regarding the utility of other therapies commonly available in high-resource settings is difficult. For example, are intravenous fluid boluses safe in the absence of close patient monitoring and respiratory support equipment? Are central venous catheters beneficial for guiding volume resuscitation in a resource-limited setting when weighed against the attendant risks of mechanical complications and infection? Given global efforts to improve oxygen monitoring capacity and supply, what level of oxygen should be targeted in septic patients? Furthermore, it is not known if (or which) isolated interventions in sepsis, unbundled from the packages of sepsis care that have been adopted in high resource hospitals, may still result in net benefit.

The etiologies of sepsis in many resource-limited environments differ from high resource settings or have not been well described. Bacteraemia studies implicate a wide range of pathogens [10,32-34]. Many other organisms including *Plasmodium falciparum*, dengue virus, influenza, *Mycobacterium tuberculosis*, *Cryptococcus neoformans* and *Rickettsia* species may cause severe clinical illness that cannot be easily distinguished from typical bacterial sepsis [10,35-39]. In addition, immune suppression from HIV is an important risk factor for adult sepsis in places where HIV prevalence is high [10]. Thus, clinical trials of sepsis therapies conducted in high-resource settings and targeting mostly bacterial infection may not be generalizable to resource-limited settings. This issue is further compounded by the dearth of diagnostic capacity to identify the specific etiologies of sepsis and to determine antimicrobial susceptibility in most district hospitals. Consequently, there is little empirically-derived local or regional data to guide hospital procurement and clinician selection of appropriate antimicrobial treatment.

Finally, populations in resource-limited settings are likely to differ from those in high-resource settings in a variety of ways. Nutritional status, co-morbidities, health seeking behaviors, access to health care, and use of over-the-counter and sub-standard or counterfeit antimicrobials may all influence the mode of presentation that can impact clinical outcome. Thus, management strategies developed in high-resource populations may not be directly applicable in resource-limited populations.

An excellent example of the risk of extrapolating principles of sepsis care across settings was demonstrated by the recent FEAST trial [40]. This large trial involving more than 3,000 African children with severe acute infections showed that children receiving fluid boluses had a higher mortality than children receiving no fluid bolus. This unexpected finding, which arguably may be more generalizable to adults in the same setting than findings from studies performed in developed countries, suggests that validation studies are required to establish the benefit of the IMAI approach which is largely based on lower level evidence. Furthermore, mortality among all patients enrolled in the FEAST trial was strikingly lower (9.4%) than in severely ill children enrolled in previous studies from similar settings (28.2%) [41]. This effect may be attributable to targeted training of all health staff on triage and monitoring prior to commencement of the study and supports the importance of emergency care training of health workers.

### A sepsis management pathway in resource-limited settings

Starting in April 2009, a working group was convened by the WHO to develop sepsis management guidelines in resource-limited settings. The group comprised 18 international experts from five continents trained in internal medicine, emergency medicine, anesthetics, critical care, infectious disease and pulmonology. Three face-to-face meetings (one co-incident with the IMAI DCM pulmonary and emergency expert groups meeting), evidence review and numerous email consultations were held. Development of the guidelines occurred by consensus and recommendations were not necessarily restricted by the lack of a high quality evidence base. These efforts eventually dovetailed with the IMAI DCM development process (in development since 2006) and in November 2009, the sepsis guidelines were adapted for management of severely ill patients during the H1N1 influenza pandemic [42]. In July 2011, prior to its final approval for publication, the WHO held a three-day external review of the IMAI DCM which includes the sepsis guidelines for resource-limited settings.

**Figure 1:**
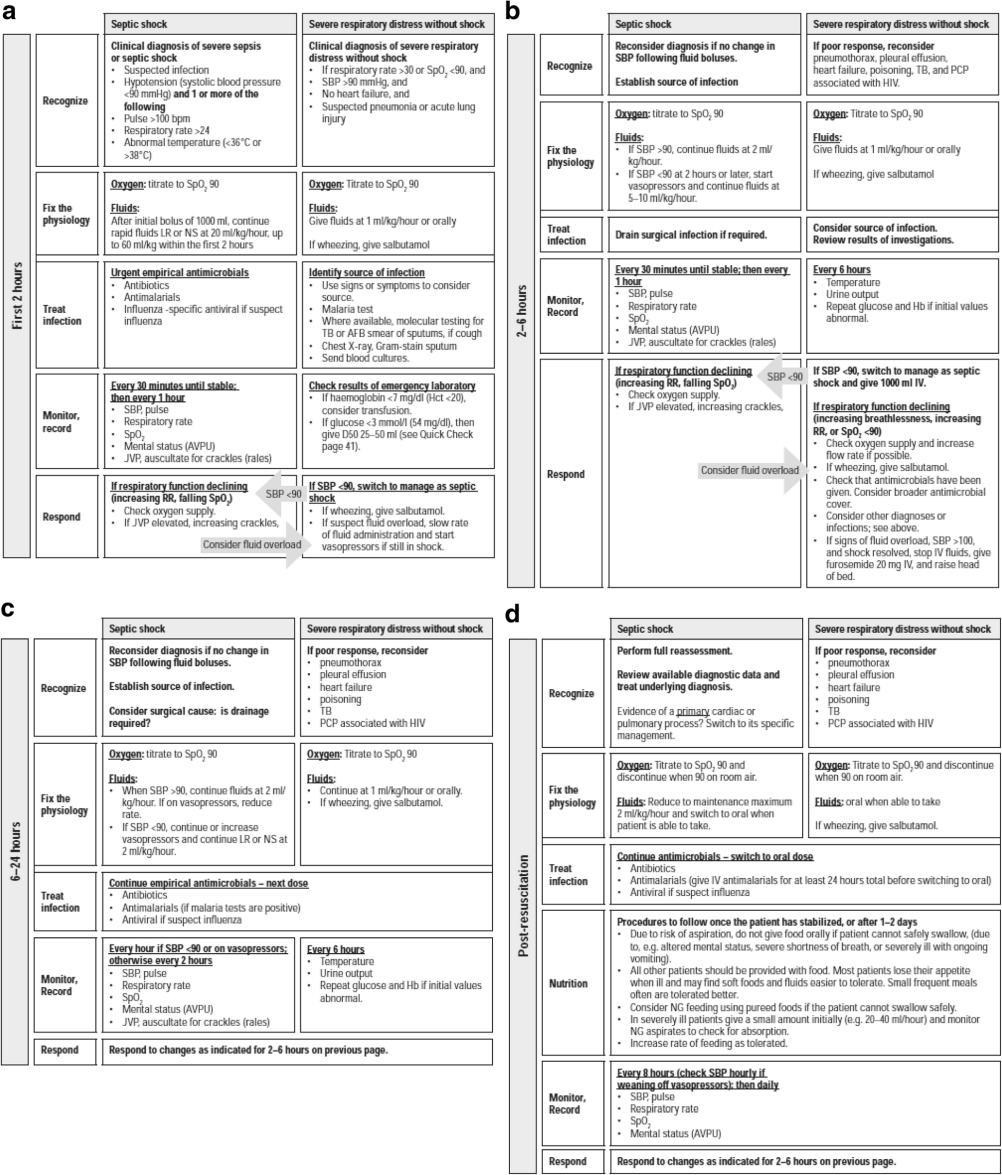
**Guidelines for the management of septic shock and severe respiratory distress without shock in resource-limited settings.** (**a**) Algorithm for the first 2 hours of hospitalization. (**b**) Algorithm for hours 2 to 6 after hospitalization. (**c**) Algorithm for hours 6 to 24 after hospitalization. (**d**) Algorithm for the post-resuscitation period. (Permission granted by the World Health Organization for reproducing the contents of this figure from the IMAI District Clinician Manual [2].)

The guidelines take into consideration potential resource constraints, acknowledging that much of the world does not have access to advanced diagnostics or resuscitative techniques. They provide instruction on sepsis management in resource-limited environments throughout a patient’s hospitalization course with emphasis on the first 2 hours (Figure 1a), 2 to 6 hours (Figure 1b), 6 to 24 hours (Figure 1c) and post-resuscitation periods (Figure 1d) [2]. The guidelines call for the early recognition of two of the most important indicators of organ dysfunction in sepsis: hypotension and acute respiratory distress. Key management principles reiterated through the guidelines include early recognition, treating infection early and broadly while establishing source control and fixing physiologic aberrations by optimizing tissue perfusion with judicious fluids and oxygen. In addition, the guidelines emphasize regular and frequent monitoring of the patient's response to treatment and a plan for appropriate action based on changes in clinical status. In light of the risk of volume overloading patients with possible acute lung injury/acute respiratory distress syndrome, the guidelines specify more conservative fluids for such patients (1 ml/kg/hour intravenously or orally) and other appropriate modifications in the resuscitation strategy. Further guidance on the management of conditions which may deviate from these guidelines is provided in the DCM, including a more conservative fluid management strategy for dengue; careful attention to possible development of fluid overload/pulmonary edema in severe malaria; specific antimicrobial recommendations for malaria, tuberculosis, maternal sepsis-related conditions (for example, amnionitis, post-partum sepsis and septic abortion) and certain viral hemorrhagic fevers (such as Lassa fever).

Most importantly, these guidelines do not exist within a vacuum. They are situated within a larger context of expedited assessment, triage and immediate intervention, using algorithms that rapidly differentiate life threatening emergency conditions from those that require less urgent care. Thus, all patients are similarly triaged by assessing whether they exhibit emergency signs related to the airway and breathing (obstruction, central cyanosis, severe respiratory distress), circulation (weak or fast pulse, capillary refill longer than three seconds, heavy bleeding or severe trauma), altered level of consciousness or convulsions or pain from a life-threatening cause [43]. The sepsis management algorithm is only triggered once underlying infection is suspected and other causes of circulatory impairment have been excluded. To avoid inappropriate guideline implementation, emphasis is placed on supporting clinical reasoning and use of differential diagnosis tables. Ultimately, patients are treated for the most likely and urgent disease syndromes without premature exclusion of alternative diagnoses, using methods and resources available even in resource-limited settings.

Successful sepsis algorithm implementation depends, above all, on improving health worker capacity to manage severely ill patients through effective training to use the algorithm as well as constant revision through new research. Accordingly, a district hospital training program based on the DCM to improve the capacity for triage and management of severe acute illness focused on septic shock and respiratory distress has been piloted by the WHO in Uganda, Malawi, Ethiopia and Rwanda. In addition to the principles of triage and management, the training also emphasizes the importance of achieving clinical management benchmarks using targeted clinical performance measures based on reasonable standards of care in resource-limited settings. Also, simplified data collection instruments that allow bedside clinical and laboratory documentation are introduced to inform therapeutic adjustments when necessary and facilitate long-term quality improvement and clinical research efforts. A similar training program has already been shown to reduce mortality in pediatric patients in a resource-limited setting [44].

### Limitations, knowledge gaps and future directions

The proposed sepsis pathway is based on a sound physiological rationale and is derived from the best evidence currently available pertinent to the care of adults with sepsis, most of which was developed in high resource settings. A subsequent independent evidence-based review of sepsis management in resource-limited settings arrived at very similar recommendations and confirmed that evidence from resource-limited settings is deplorably scarce [45]. Despite the lack of empirically derived data in resource-limited settings, we propose that it is ethically acceptable to provide guidelines to health care providers that are based on the application of sound physiological principles and the best available current evidence. It is also essential, however, that research be undertaken to evaluate areas of uncertainty in the guidelines (for example, determining the safest and most efficacious volume of fluid resuscitation for improving sepsis survival) and to validate the effectiveness of the guidelines’ performance in appropriate settings with modification of the guidelines based on these findings. This process of validating and revising guidelines mirrors the approach used in high resource settings by the Surviving Sepsis Campaign [46]. Given the unique challenges of infrastructure and human capacity limitations and the substantial knowledge gaps specific to resource-limited settings, key issues for further investigation include:

1. Evaluating the efficacy and effectiveness of training in the proposed management pathway for improving patient outcomes and health care worker performance, particularly in patient populations and settings where invasive monitoring and extensive laboratory investigations are absent.

2. Improving the recognition and defining the natural history of sepsis caused by organisms endemic in developing countries, while refining management of those clinical syndromes where a pathogen-specific guideline may be superior. Further clinical research is necessary to determine where and how the proposed guidelines can complement existing disease-specific recommendations and whether specific components require further evaluation.

3. Through the evaluation of severity scoring systems and differing clinical definitions, defining groups for which early intervention may be either particularly beneficial or futile.

4. Determining the potential benefit of adjunctive treatments (such as non-invasive ventilation) and interventions that may augment current care (for example, the use of oral fluids, the use of ultrasound to guide amount of fluid resuscitation, the role of early feeding and micronutrient supplementation and the training of patient relatives as clinical care attendants).

5. Conducting relative cost-benefit analyses addressing supply issues and resource utilization for basic treatments, such as oxygen, intravenous fluids and antimicrobials can aid health facilities in determining how to prioritize resources required for sepsis management.

6. Tailoring treatment more effectively by enhancing on-site diagnosis, through either improved basic microbiology services, expanded sentinel surveillance microbiologic laboratory sites or the use of novel rapid diagnostic tests comprising assays which can identify sepsis etiology and/or provide prognostic information at the point of care.

## Conclusion

Current evidence suggests that the mortality and morbidity of severe sepsis can be improved by effective clinical interventions applied in a timely and systematic manner. In resource-limited settings where the burden of disease is highest but least well studied, there remain many barriers to the adoption of such practices. The essential elements of care of critically ill patients in resource-limited settings are a triage system that identifies critical illness quickly, appropriate protocols for managing common medical emergencies, the availability of requisite interventions, targeted training of providers in critical care principles, and a data collection and quality management system to monitor implementation and impact. This sepsis management pathway for resource-limited settings, a subset of the larger severe illness recognition and management algorithms within the IMAI District Clinician Manual, adheres to these principles and provides an essential first step in improving outcomes. It should be feasible to design and implement a package comprising these elements adapted for any setting but in order to ensure that such guidelines fulfill their potential for saving lives, a robust global effort championing training and research in the management of acute severe illnesses, such as sepsis, in resource-limited settings is essential.

## Abbreviations

IMAI: Integrated management of adolescent and adult illness; DCM: District Clinician Manual; WHO: World health organization.

## Competing interests

The authors declare they have no competing interests.

## Authors’ contributions

STJ, ML and TEW drafted the manuscript. All authors contributed to the development of the sepsis guidelines, provided technical edits for revisions of the manuscript and read and approved the final manuscript.

## Authors’ information

All authors were part of World Health Organization working groups to develop guidelines for the management of severe sepsis in resource-limited settings. In their previous roles at the World Health Organization, CR and ML led the activities of this working group. During the development of these sepsis guidelines and the District Clinician Manual, SG was the team leader for the IMAI program in the HIV department at the World Health Organization. STJ, HC and SG were overall technical editors for the IMAI District Clinician Manual.

STJ and ML are co-first authors.

## Pre-publication history

The pre-publication history for this paper can be accessed here:

http://www.biomedcentral.com/1741-7015/11/107/prepub
